# A Mobile-Based Intervention for Glycemic Control in Patients With Type 2 Diabetes: Retrospective, Propensity Score-Matched Cohort Study

**DOI:** 10.2196/15390

**Published:** 2020-03-11

**Authors:** Jing Li, Li Sun, Yaogang Wang, Lichuan Guo, Daiqing Li, Chang Liu, Ning Sun, Zheng Xu, Shu Li, Yunwen Jiang, Yuan Wang, Shunming Zhang, Liming Chen

**Affiliations:** 1 National Health Commission Key Laboratory of Hormones and Development, Tianjin Key Laboratory of Metabolic Diseases Chu Hsien-I Memorial Hospital & Tianjin Institute of Endocrinology Tianjin Medical University Tianjin China; 2 School of Nursing Tianjin Medical University Tianjin China; 3 School of Public Health Tianjin Medical University Tianjin China; 4 Yiducloud Technologies Co, Ltd Beijing China; 5 Yu-Tang Department iHealth Labs Inc Beijing China

**Keywords:** mobile health, glycemic control, type 2 diabetes, propensity score matching

## Abstract

**Background:**

Mobile-based interventions appear to be promising in ameliorating huge burdens experienced by patients with type 2 diabetes. However, it is unclear how effective mobile-based interventions are in glycemic management of patients with type 2 diabetes based on real-world evidence.

**Objective:**

This study aimed to evaluate the effectiveness of a mobile-based intervention on glycemic control in patients with type 2 diabetes based on real-world population data.

**Methods:**

This retrospective, propensity score-matched cohort study analyzed longitudinal data from a clinical electronic health database. The study population included 37,913 patients with type 2 diabetes at cohort entry between October 1, 2016, and July 31, 2018. A total of 2400 patients were matched 1:1, using propensity score matching, into the usual care and mobile health (mHealth) groups. The primary outcomes of glycemic control included control rates of glycated hemoglobin (HbA_1c_), fasting blood glucose (FBG), and postprandial 2-hour blood glucose (P2BG). Mean values and variation trends of difference with 95% CI were the secondary outcomes. The general linear model was used to calculate repeated-measures analyses of variance to examine the differences between the two groups. Subgroup and sensitivity analyses were performed.

**Results:**

Of the 2400 patients included in the analysis, 1440 (60.00%) were male and the mean age was 52.24 years (SD 11.56). At baseline, the control rates of HbA_1c_, FBG, and P2BG in the mHealth and usual care groups were 45.75% versus 47.00% (*P*=.57), 38.03% versus 32.76% (*P*=.07), and 47.32% versus 47.89% (*P*=.83), respectively. At the 3-, 6-, 9-, and 12-month follow-ups, the mHealth group reported higher control rates of HbA_1c_ than did the usual care group: 69.97% versus 46.06% (*P*<.001), 71.89% versus 61.24% (*P*=.004), 75.38% versus 53.44% (*P*<.001), and 72.31% versus 46.70% (*P*<.001), respectively. At the four follow-up sessions, the control rates of FBG in the mHealth and usual care groups were statistically different: 59.24% versus 34.21% (*P*<.001), 56.61% versus 35.14% (*P*<.001), 59.54% versus 34.99% (*P*<.001), and 59.77% versus 32.83% (*P*<.001), respectively. At the four follow-up sessions, the control rates of P2BG in the mHealth group were statistically higher than in the usual care group: 79.72% versus 48.75% (*P*<.001), 80.20% versus 57.45% (*P*<.001), 81.97% versus 54.07% (*P*<.001), and 76.19% versus 54.21% (*P*=.001), respectively. At the four follow-up sessions, the percentages of HbA_1c_ reduction in the mHealth group were 8.66% (95% CI 6.69-10.63), 10.60% (95% CI 8.66-12.54), 10.64% (95% CI 8.70-12.58), and 8.11% (95% CI 6.08-10.14), respectively. At the four follow-up sessions, the percentages of P2BG reduction in the mHealth group were 8.44% (95% CI 7.41-10.73), 17.77% (95% CI 14.98-20.23), 16.23% (95% CI 13.05-19.35), and 16.91% (95% CI 13.17-19.84), respectively. Starting from the sixth month, the mean HbA_1c_ and P2BG values in the two groups increased slightly.

**Conclusions:**

This mobile-based intervention delivered by a multidisciplinary team can better improve glycemic control rates of patients with type 2 diabetes than usual care. These effects were best sustained within the first 6 months. Starting from the sixth month, intensive management needs to be conducted to maintain long-term effectiveness of the mobile-based intervention.

## Introduction

The number of adults with diabetes worldwide increased from 108 million to 422 million between 1980 and 2014 [1], with a projected increase to 642 million by 2040 [2]. In China, the overall prevalence of diabetes in the adult population was estimated to be 11.6% in 2010 and was less than 1.0% in 1980 [3]. Patients with type 2 diabetes mellitus account for 90%-95% of those with diabetes [4]. Diabetes not only results in blindness, cardiovascular disease, kidney failure, and other long-term consequences that substantially impact quality of life and years of life lived with disability [5,6], but also increases the risk of cancer and all-cause mortality [7-11]. Therefore, the prevention and control of diabetes is becoming more and more important.

For people with diabetes, a series of cost-effective interventions can improve their health outcomes, regardless of what type of diabetes they may have [12-17]. These interventions mainly include glycemic control, combined with diet, physical activity, and, if necessary, medication; control of blood pressure and lipids to reduce cardiovascular risk and other complications; and regular screening for damage to the eyes, kidneys, and feet to facilitate early treatment [12,13]. Glycemic control through quarterly physician visits with measurements of glycated hemoglobin (HbA_1c_), fasting blood glucose (FBG), and postprandial 2-hour blood glucose (P2BG) were recommended in professional treatment guidelines [14]. In addition, health education, counseling, self-management, and consistent follow-up were also important for people with diabetes [15-17]. Therefore, people with diabetes require access to systematic, ongoing, and organized care delivered by a multidisciplinary team of skilled health care providers.

Mobile health (mHealth), which is defined as the use of mobile and wireless technologies for health (ie, mobile phones or sensor technologies), aims to capitalize on the rapid uptake of information and communication technologies to improve health system efficiency and health outcomes [18-20]. This includes simple apps and complex technologies, including voice, text messaging (ie, short message service), multimedia message service, Bluetooth technology, and others [21]. These advances, combined with changing patient attitudes toward self-testing, as well as an increased interest in wearable biosensors, are enticing health care providers to shift toward the paradigm of *P4* medicine: predictive, pre-emptive, personalized, and participatory [22]. The characteristics of mobility, instantaneous access, and direct communication of mHealth allow for faster transfer of health information, which in turn supports patient management. mHealth is a promising tool for delivering interventions designed to promote lifestyle management of patients with type 2 diabetes. Use of mHealth often includes the possibility of sharing data between health professionals and their patients with diabetes, which could enhance the support to improve their self-management [23,24]. Successful use of mHealth technology requires an active user and cooperation among health professionals [25]. That is, the technology’s effectiveness is often determined by the way in which it is provided to patients or practitioners, how it is supported or taught, and how mHealth technology is added to clinical work or daily life [26].

Previous studies have shown that mobile-based interventions developed for diabetes holistic management have some effects [23-25,27,28]. A systematic review included a meta-analysis of 14 randomized trials aiming to investigate the effect of apps on HbA_1c_ in the self-management of diabetes; these studies showed that the mean reduction in HbA_1c_ among participants using an app compared with control group participants was 0.49% (95% CI 0.30-0.68; I^2^=10%) [23]. Another systematic review of high-quality review articles and meta-analysis, which focused on utilizing technology in diabetes self-management education and support services, found that technology-enabled diabetes self-management solutions significantly improved HbA_1c_ and four key elements emerged as essential for improved HbA_1c_: (1) communication, (2) patient-generated health data, (3) education, and (4) feedback [24]. Although the majority of these interventions showed improvement on primary endpoints [25,27,28], whether results will drive substantial clinical adoption is unknown because small studies, even if randomized, are unlikely to be significantly powered to demonstrate meaningful real-world effects [20]. Therefore, real-world evidence and performance data of mobile-based interventions are needed to demonstrate value or motivate stakeholder adoption.

Based on real-world population data from a clinical electronic health database, this study aimed to evaluate the effectiveness of a mobile-based intervention on glycemic control in patients with type 2 diabetes and to explore the change in trends of glycemic parameters in the short and long term.

## Methods

### Study Design

We conducted a retrospective, propensity score-matched cohort study using electronic health data from a clinical database in Tianjin, China. This clinical database was established in June 2014. The database contained longitudinal outpatient records of patients in five primary care practices and one tertiary care hospital specializing in diabetes, including their demographics, primary and secondary diagnoses, and clinical examination results.

### Cohort Selection

**Figure 1:**
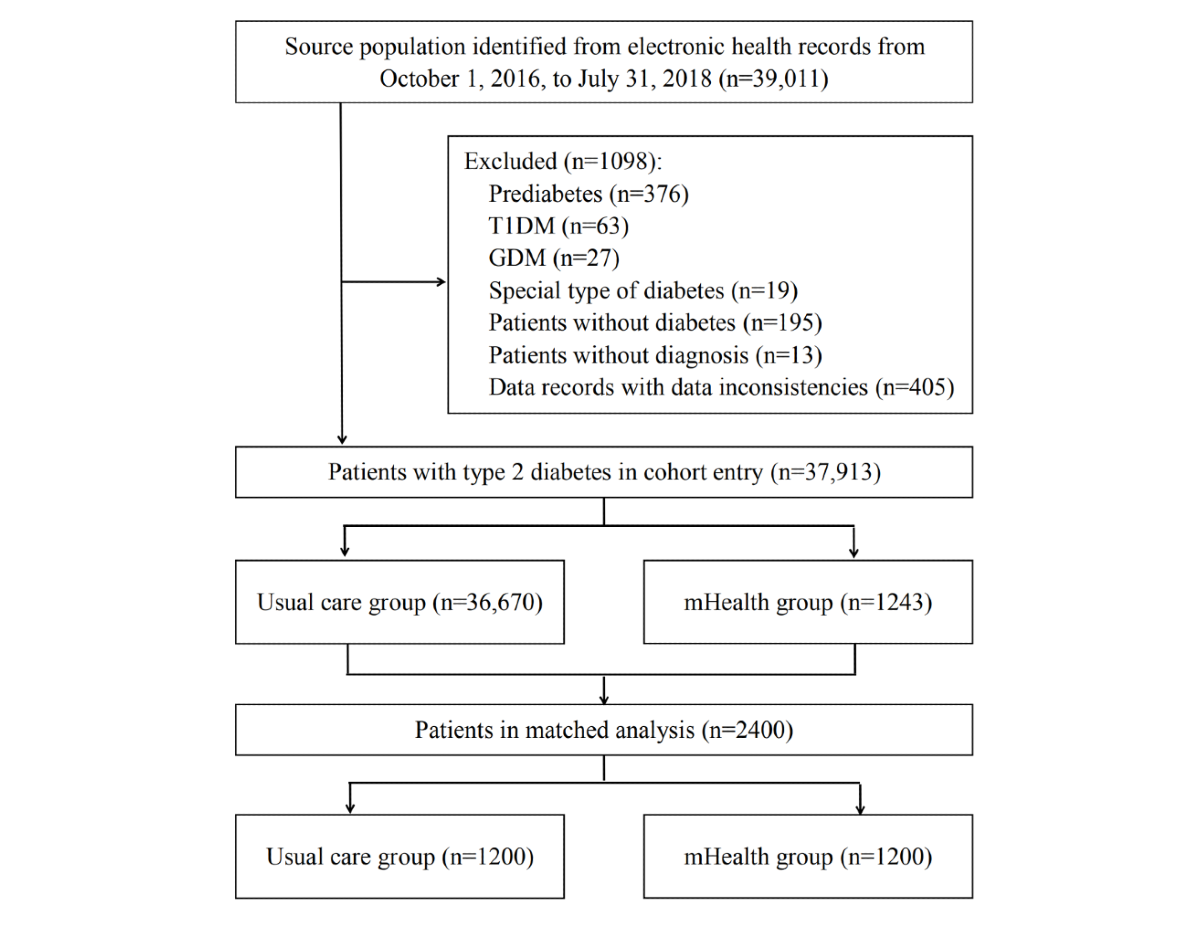
Flowchart for cohort selection. GDM: gestational diabetes mellitus; T1DM: type 1 diabetes mellitus.

The study cohort included 37,913 patients with type 2 diabetes who were registered in this clinical database between October 1, 2016, and July 31, 2018. The flowchart for cohort selection is shown in Figure 1.

All of the patients who were registered in the clinical database between October 1, 2016, and July 31, 2018, were included in our source population. In the end, we identified 39,011 individuals with 1,793,841 records from the clinical database. The unique ID numbers were used to identify the records of the patients with different outpatient numbers in different clinical settings. Using Microsoft Visual Basic 6.0, we developed sophisticated applications to extract and filter these data. After source population selection, the following exclusion criteria were applied: (1) prediabetes, (2) type 1 diabetes, (3) gestational diabetes, (4) special type of diabetes, (5) patients without diabetes, (6) patients without diagnosis, and (7) data records with data inconsistencies. Finally, we identified 37,913 patients with type 2 diabetes.

This unmatched cohort was divided into two groups, including the usual care group (n=36,670) and the mHealth group (n=1243). This was an observational study originating from the real world with no constraints on the cohort entry of participants in either group.

### Interventions

The usual care group received standard medical care for patients with type 2 diabetes. Every 3 months, patients in this group underwent regular reviews to re-examine HbA_1c_, FBG, and P2BG levels. These lab examination results were considered as evidence to support doctors’ decisions to adjust medications. Meanwhile, patients in this group received routine health education at each session.

In addition to usual care, the mHealth group received a mobile-based intervention, which was continuous, real-time, personalized health care delivered by a multidisciplinary team consisting of doctors, nurses, health educators, and dietitians. This mobile-based intervention was based on a unified diabetes care system, which consisted of a mobile app, smart wearable medical devices (eg, wireless glucose monitor, wireless blood pressure monitor, pulse oximeter, and body composition scale), a Web platform, and a data-sharing cloud platform. Patients with type 2 diabetes in the mHealth group followed new flows in the clinical settings (see Multimedia Appendix 1). Patients’ management and education in the mHealth group extended from the clinic to home. They used the wireless glucose monitors and app to perform glucose checks at home. When they experienced hypoglycemia or hyperglycemia, the app could provide tips to help them regulate their glucose levels. Patients also sent their results immediately to the support team about what to do. The care team and the service support team members would be notified when the patient was experiencing abnormal glucose levels. They then phoned the patient to inquire about their recent medication, diet, and exercise, and to help the patient in analyzing possible reasons for the abnormal glucose level. If necessary, they would invite the patient for further in-clinic consultation or guide the patient to adjust their diet or exercise by phone. Patients could also record their meals in the app and get feedback from the service support team. According to an image or a description of the food, the team would provide an overall rating of the meal, comments on portion and nutrition, and suggestions on how to do better. Patients could log their exercise type and duration into the app. The service support team created updated knowledge covering blood glucose, blood pressure, food, fitness, oral medication, insulin, psychology, and complications in the form of articles, videos, and attractive posters. Patients had access to this educational information whenever and wherever possible.

### Outcome Definition

The primary outcomes were control rates of HbA_1c_, FBG, and P2BG at baseline and at 3-, 6-, 9-, and 12-month follow-ups. We identified the control rates according to guidelines for the prevention and control of type 2 diabetes in China [29]. Control objectives were defined as follows: (1) HbA_1c_ <7%, (2) 4.4 mmol/L< FBG <7.0 mmol/L, and (3) P2BG <10 mmol/L. We also considered mean values and variation trends of difference (VTD) with 95% CI, separately, as secondary outcomes. The formula for calculating VTD was as follows:

VTD = (Value_n_ - Value_baseline_)/Value_baseline_ × 100%

where Value_n_ and Value_baseline_ denoted the sample values of HbA_1c_, FBG, and P2BG at *n*-month (n=3, 6, 9, and 12) follow-up and baseline, respectively [30]. If VTD was positive, it represented the percentage of increase; if VTD was negative, it represented the percentage of reduction.

### Covariates

Demographic and chronic disease covariates included sex, age, comorbidity (ie, hyperlipidemia and hypertension), P2BG, FBG, HbA_1c_, and low-density lipoprotein (LDL) cholesterol. App use-related covariates included times of FBG and P2BG self-testing, diet records, exercise records, and out-of-hospital follow-up.

### Statistical Analysis

**Figure 2:**
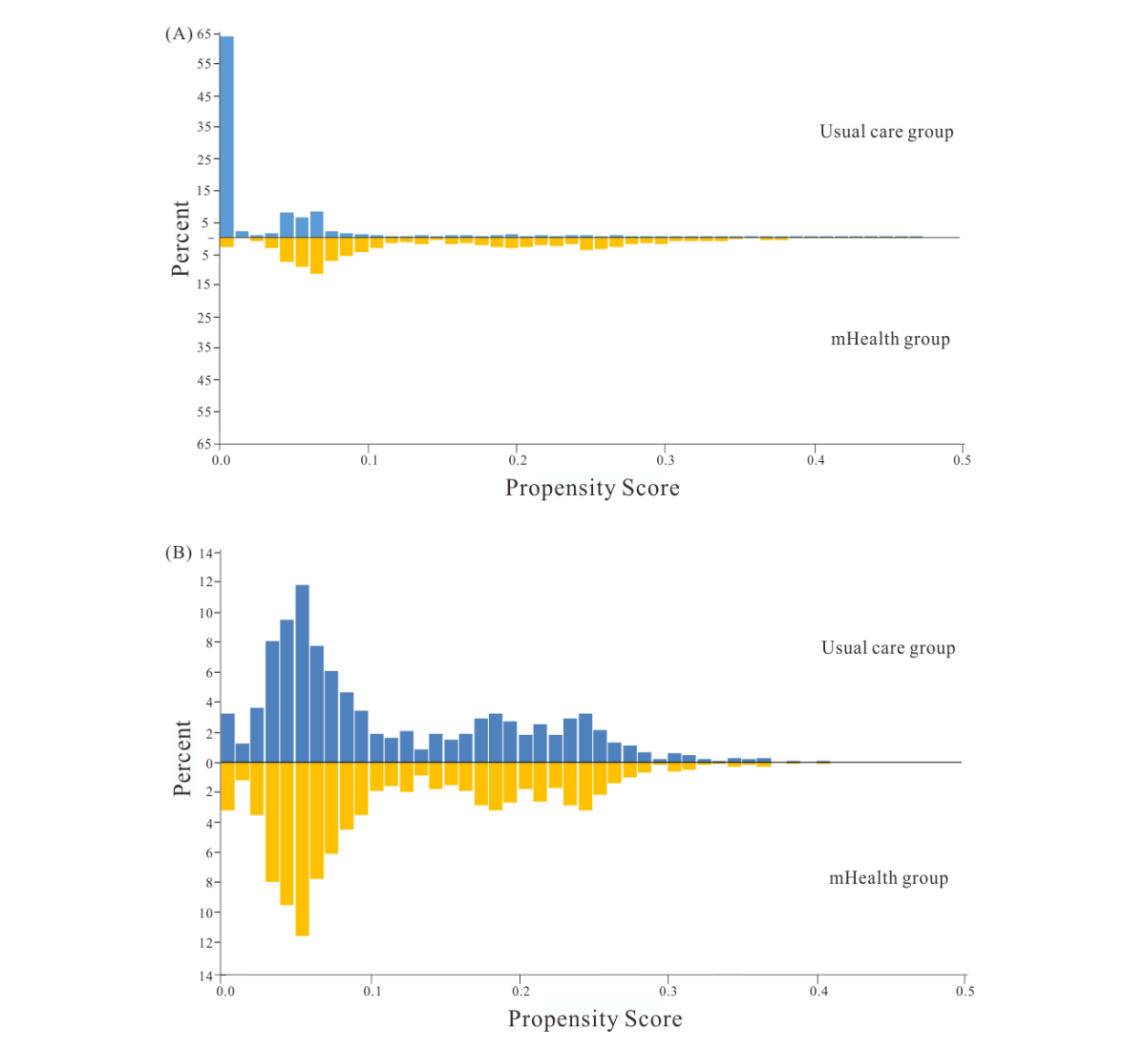
Mirror histograms. (A) Before match. (B) After match.

To control for the nonrandom assignment of patients, a logistic regression model that predicted the likelihood of being included in the mHealth group was constructed and used as the propensity score. Patients were then matched 1:1, using propensity score matching, into the usual care group and mHealth group. We selected all of the common available variables for two-group matching [31], including sex, age group, comorbidity (ie, hyperlipidemia and hypertension), HbA_1c_ level, and LDL cholesterol level. The propensity score-matching tolerance was 0.005. No replacement was allowed, and patients were matched only once. Standardized differences with mirror histograms before and after matching are shown in Figure 2 and Multimedia Appendix 2. We evaluated the balances of matched covariates with standardized differences [29] and considered differences of less than 10% to be matched sufficiently [32,33].

We presented categorical variables as numbers (percentages) and continuous variables as means (SDs) or 95% CIs, or as medians (IQRs), as appropriate. Descriptive statistics were used to analyze the patient demographics. Binary or categorical outcome measures were analyzed using the chi-square test and continuous measures were analyzed using the *t* test or a nonparametric equivalent (eg, Wilcoxon rank test). We used the general linear model to calculate repeated-measures analyses of variance to examine mean differences of two groups at baseline and at 3-, 6-, 9-, and 12-month follow-ups. Subgroup analyses to explore the effects of this mobile-based intervention in different patient subgroups were undertaken for the primary and secondary outcomes. The subgroups, specified in the statistical analysis, included patient demographics: sex, age group, hyperlipidemia, and hypertension. The total proportion of missing values at the 12-month follow-up was 12.3%. The proportions of missing data at each data point were 4.5% (3-month follow-up), 7.1% (6-month follow-up), and 9.5% (9-month follow-up). Expectation maximization was used to estimate the missing values of continuous variables. A sensitivity analysis was performed by repeating our primary analysis but excluding patients with hyperlipidemia or hypertension.

We determined statistical significance using a two-tailed *P* value of less than .05. All of the statistical analyses were carried out using SPSS Statistics for Windows, version 25.0 (IBM Corp).

## Results

### Patient Demographics

Of the 39,011 patients, 37,913 met the selection criteria for additional analysis (see Figure 1). In the unmatched cohort, the proportion of male patients was 53.41% (20,248/37,913), patients’ mean age was 57.94 years (SD 12.10), and 88.13% of patients were 36-74 years of age. The proportion of patients with hyperlipidemia was 20.52% (7779/37,913), and the proportion of patients with hypertension was 8.11% (3073/37,913). The mean HbA_1c_ level was 7.86% (SD 1.25) and the mean LDL cholesterol level was 3.37 mmol/L (SD 0.65). Table 1 shows the baseline demographics of patients with type 2 diabetes in unmatched and propensity score-matched cohorts. There were significant differences in demographics or glycemic parameters between the usual care group and the mHealth group.

A propensity score match was then performed and 2400 patients were matched 1:1. After matching, covariates were well balanced and we did not observe any significant differences between groups (see Table 1). In the propensity score-matched cohort, the proportion of male patients was 60.00% (1440/2400), the mean age of patients was 52.24 years (SD 11.56), and 90.13% of patients were 36-74 years of age. A total of 48.54% (1165/2400) of patients had a comorbidity of hyperlipidemia, and 44.38% (1065/2400) of patients had a comorbidity of hypertension. The mean HbA_1c_ level was 7.76% (SD 1.39) and the mean LDL cholesterol level was 3.27 mmol/L (SD 0.79).

**Table 1:** Baseline demographics of patients with type 2 diabetes in unmatched and propensity score-matched cohorts.

Characteristic		Unmatched cohort				Propensity score-matched cohort			
		mHealth group (n=1243)	Usual care group (n=36,670)	*P* value	Std diff^a^	mHealth group (n=1200)	Usual care group (n=1200)	*P* value	Std diff
**Sex, n (%)**									
	Male	777 (62.51)	19,471 (53.10)	<.001	19.13	738 (61.50)	702 (58.50)	.15	3.80
	Female	466 (37.49)	17,199 (46.90)			462 (38.50)	498 (41.50)		
**Age group (years), n (%)**									
	≤35	80 (6.44)	2086 (5.69)	<.001	29.17	73 (6.08)	96 (8.00)	.07	7.31
	36-59	657 (52.85)	15,914 (43.40)		19.10	631 (52.58)	634 (52.83)		0.40
	60-74	464 (37.33)	16,379 (44.66)		15.10	454 (37.84)	444 (37.00)		1.67
	≥75	42 (3.38)	2291 (6.25)		13.13	42 (3.50)	26 (2.17)		7.78
**Comorbidity, n (%)**									
	Hyperlipidemia	647 (52.05)	7132 (19.45)	<.001	72.67	604 (50.33)	561 (46.75)	.09	7.00
	Hypertension	593 (47.71)	2480 (6.76)	<.001	102.25	550 (45.83)	515 (42.92)	.16	5.80
**Biochemical indicator, mean (SD)**									
	HbA_1c_^b^ level (%)	7.82 (1.60)	7.86 (1.24)	<.001	3.16	7.83 (1.60)	7.70 (1.15)	.26	9.35
	LDL^c^ cholesterol (mmol/L)	3.26 (0.88)	3.38 (0.64)	<.001	15.10	3.29 (0.88)	3.25 (0.69)	.71	5.06

^a^Std diff: standardized difference.

^b^HbA_1c_: glycated hemoglobin.

^c^LDL: low-density lipoprotein.

Until July 31, 2018, the total number of times of starting up the app, self-monitoring of glycemic parameters, diet recording, exercise recording, and out-of-hospital follow-ups were 80,129; 172,355; 17,860; 4464; and 5264, respectively; the median follow-up time was 457 days.

### Control Rates of Glycemic Parameters

**Figure 3:**
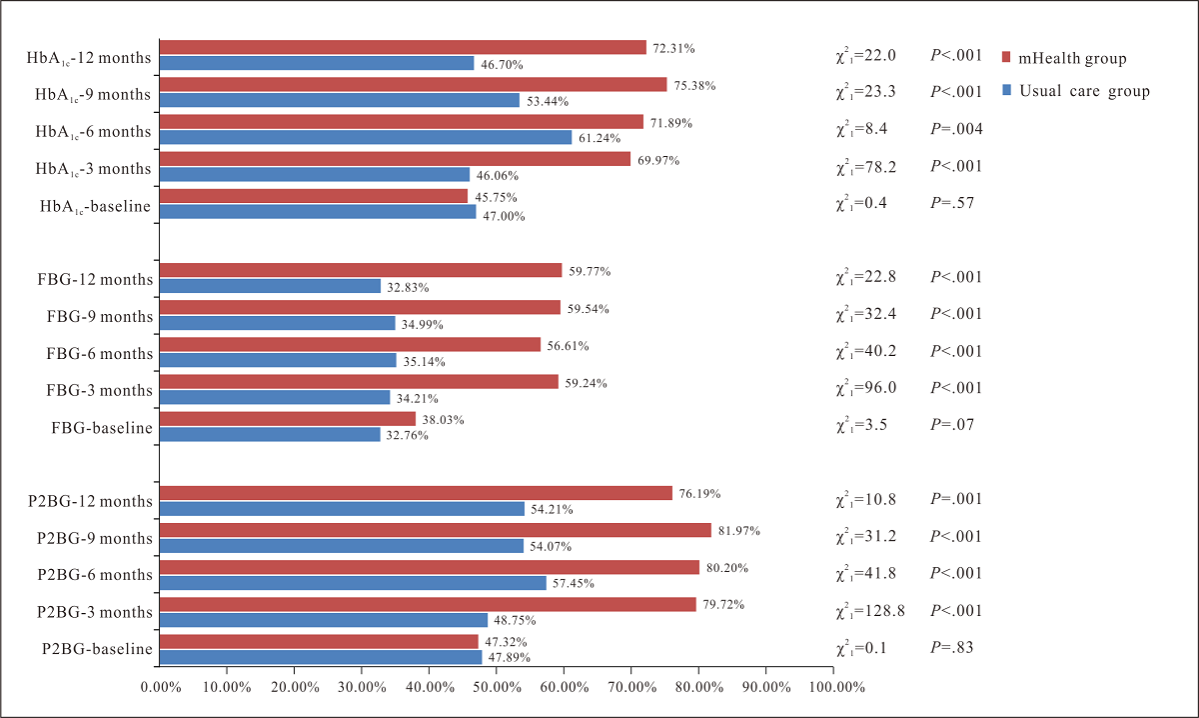
Control rates of glycated hemoglobin (HbA_1c_), fasting blood glucose (FBG), and postprandial 2-hour blood glucose (P2BG) in the mHealth and usual care groups at different follow-up sessions.

At baseline, the control rates of HbA_1c_, FBG, and P2BG in the mHealth and usual care groups were 45.75% versus 47.00% (*P*=.57), 38.03% versus 32.76% (*P*=.07), and 47.32% versus 47.89% (*P*=.83), respectively. The control rates of HbA_1c_, FBG, and P2BG in both groups at different follow-up sessions are shown in Figure 3.

At the 3-, 6-, 9-, and 12-month follow-ups, the mHealth group reported higher control rates of HbA_1c_ than usual care, which were 69.97% versus 46.06% (*P*<.001), 71.89% versus 61.24% (*P*=.004), 75.38% versus 53.44% (*P*<.001), and 72.31% versus 46.70% (*P*<.001), respectively. Differences in the control rates between the two groups at these four follow-up sessions were 23.91%, 10.65%, 21.94%, and 25.61%, respectively. At the 9-month follow-up, the mHealth group reported the highest control rate of HbA_1c_, which was 75.38%.

Additionally, at the 3-, 6-, 9-, and 12-month follow-ups, the control rates of FBG in the mHealth and usual care groups were statistically different, which were 59.24% versus 34.21% (*P*<.001), 56.61% versus 35.14% (*P*<.001), 59.54% versus 34.99% (*P*<.001), and 59.77% versus 32.83% (*P*<.001), respectively. The control rates of P2BG in the mHealth group were statistically higher than in the usual care group, which were 79.72% versus 48.75% (*P*<.001), 80.20% versus 57.45% (*P*<.001), 81.97% versus 54.07% (*P*<.001), and 76.19% versus 54.21% (*P*=.001), respectively.

### Mean Values of Glycemic Parameters

Table 2 shows the effects of this mobile-based intervention on glycemic parameters. The mean values of HbA_1c_, FBG, and P2BG in the mHealth group were significantly lower than those in the usual care group at the 3-, 6-, 9-, and 12-month follow-ups (*P*<.01). The *P* values of the *month* factor were less than .001, which meant that the HbA_1c_, FBG, and P2BG levels changed with time. The *group* and *month* factors had interaction effects in the mean values of HbA_1c_, FBG, and P2BG (*P*<.01), which meant that the effect of the *time* factor varied with the *group*. These results identified improved effects due to this mobile-based intervention on changes of the HbA_1c_, FBG, and P2BG mean values.

Multimedia Appendices 3-7 show that, compared with usual care, at the 3-, 6-, 9-, and 12-month follow-ups, both sexes in the mHealth group reported significantly lower mean values of HbA_1c_, FBG, and P2BG (*P*<.05), although we did not observe any significant differences in the P2BG mean values of female participants between the two groups at the 12-month follow-up (*P*=.20). Patients aged 36-74 years in the mHealth group had steadily lower HbA_1c_, FBG, and P2BG mean values than those in the usual care group (*P*<.05). No statistically significant difference was observed in P2BG mean values of patients aged 36-59 years between the two groups at the 12-month follow-up (*P*=.09). Patients younger than 35 or older than 75 years of age in the mHealth group reported unstable variation trends of mean values.

**Table 2:** Effects of the mobile-based intervention on glycemic parameters.

Variables and group		Measurement session					F months	*P* value	F groups	*P* value	F months × group	*P* value
		Baseline	3 months	6 months	9 months	12 months						
**HbA_1c_^a^ (%), mean (SD)**												
	mHealth group	7.83 (1.60)	6.70 (0.73)	6.60 (0.61)	6.44 (0.59)	6.75 (0.76)	11.822	<.001	0.058	.08	5.905	.003
	Usual care group	7.70 (1.15)	7.36 (1.17)	6.82 (0.64)	6.97 (0.63)	7.12 (0.64)						
	Z	-1.123	-20.382	-18.592	-19.922	-9.657						
	*P* value	.26	<.001	<.001	<.001	<.001						
**FBG^b^ (mmol/L), mean (SD)**												
	mHealth group	8.34 (2.41)	6.51 (1.27)	6.74 (2.11)	6.68 (1.49)	6.89 (1.52)	9.614	<.001	16.425	<.001	5.762	<.001
	Usual care group	8.68 (2.34)	8.53 (2.37)	8.45 (2.45)	8.38 (2.39)	8.47 (2.68)						
	Z	-1.326	-15.268	-17.315	-14.831	-5.755						
	*P* value	.20	<.001	<.001	<.001	<.001						
**P2BG^c^ (mmol/L), mean (SD)**												
	mHealth group	11.14 (4.40)	8.03 (1.75)	7.75 (1.90)	7.89 (1.77)	8.29 (2.38)	12.424	<.001	9.566	.002	7.193	<.001
	Usual care group	10.36 (2.94)	9.99 (2.99)	9.85 (2.56)	9.93 (2.83)	9.97 (2.91)						
	Z	-1.575	-23.995	-20.921	-16.243	-3.149						
	*P* value	.12	<.001	<.001	<.001	.002						

^a^HbA_1c_: glycated hemoglobin.

^b^FBG: fasting blood glucose.

^c^P2BG: postprandial 2-hour blood glucose.

### Variation Trends of Difference for Glycemic Parameters

**Figure 4:**
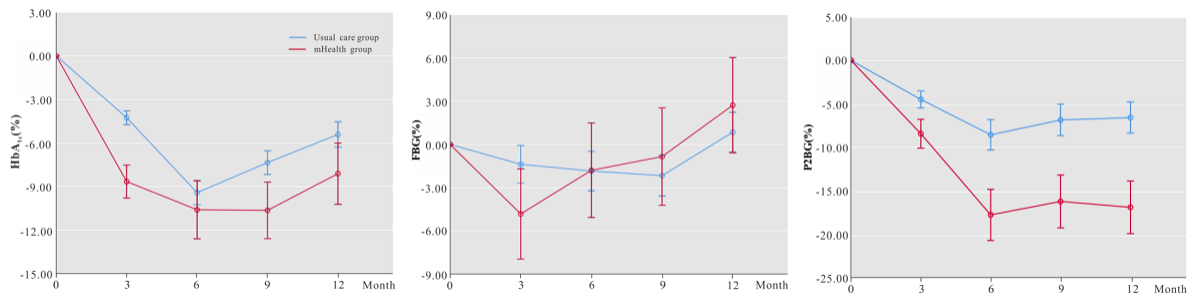
Variation trends of glycated hemoglobin (HbA_1c_), fasting blood glucose (FBG), and postprandial 2-hour blood glucose (P2BG) differences.

Figure 4 shows the variation trends of difference for glycemic parameters between the two groups. At the 3-, 6-, 9-, and 12-month follow-ups, the percentages of HbA_1c_ reduction in mHealth group were 8.66% (95% CI 6.69-10.63), 10.60% (95% CI 8.66-12.54), 10.64% (95% CI 8.70-12.58), and 8.11% (95% CI 6.08-10.14), respectively; the percentages of P2BG reduction in the mHealth group were 8.44% (95% CI 7.41-10.73), 17.77% (95% CI 14.98-20.23), 16.23% (95% CI 13.05-19.35), and 16.91% (95% CI 13.17-19.84), respectively. Equally important was that, after 6 months, the declines in HbA_1c_ and P2BG of the two groups decreased, whereas the mHealth group experienced larger decreases in HbA_1c_ and P2BG than the usual care group. At the 3-month follow-up, the reduction of FBG in the mHealth group was larger than in the usual care group (4.83% vs 1.38%). However, starting from the sixth month, the reductions of FBG in the usual care group were larger than in the mHealth group.

### Sensitivity Analysis

We conducted sensitivity analysis, where we excluded 1587 patients with hyperlipidemia or hypertension. In the mHealth group at the baseline and the four follow-up sessions, the control rates of HbA_1c_ were 36.57%, 72.77%, 76.92%, 80.00%, and 75.00%, respectively; the control rates of FBG were 44.44%, 65.79%, 65.74%, 59.38%, and 65.63%, respectively; and the control rates of P2BG were 54.02%, 82.63%, 77.45%, 83.33%, and 72.41%, respectively. There were no significant differences between the sensitivity analysis and the primary analysis (χ^2^=10.0 *P*=.35), so we presented only the results of the primary analysis.

## Discussion

### Principal Findings

In this study, a total of 2400 patients with type 2 diabetes were matched 1:1, using propensity score matching, into the usual care and mHealth groups. A total of 60% of the patients were male and more than half were 36-59 years of age. These demographics were similar to the population in previous studies [29,34]. Our results showed the improvement in control rates of HbA_1c_, FBG, and P2BG for patients with type 2 diabetes in the mHealth group compared with those in the usual care group. These effects were best sustained within the first 6 months. Starting from the sixth month, the mean HbA_1c_ and P2BG values in the two groups increased slightly.

### Comparison With Prior Work

The role of glycemic control in preventing the development and progression of complications has been proven in diabetes [35-38], with an especially strong relationship identified between intensive glycemic control and diabetic complications and mortality. In general, a target HbA_1c_ level of less than 7% is optimal, according to diabetes guidelines [14]. Each 1% of mean HbA_1c_ value reduction has been associated with a 21% reduction in the risk of diabetes-related complications [39]. A recent study on the legacy effect of early glycemic control on future complications in type 2 diabetes showed that, compared with an HbA_1c_ of less than 6.5% for the 0-1-year early exposure period, HbA_1c_ levels of 6.5% or higher were associated with increased microvascular and macrovascular events, and HbA_1c_ levels of 7.0% or higher were associated with increased mortality [40]. However, a recent meta-analysis demonstrated that HbA_1c_ target achievement is low, with a pooled average of 43% worldwide [41], both in primary and secondary care settings. In 2013, among Chinese patients with diabetes, only 39.7% of those treated had adequate glycemic control [34]. The reason for this low target achievement, despite the expanding arsenal of glucose-lowering interventions, remains unclear [42]. Our study found that the control rates of HbA_1c_, FBG, and P2BG in the mHealth group were higher than those in the usual care group, which were much higher than the average level worldwide [41]. These findings confirmed the effectiveness of this mobile-based intervention on glycemic control in patients with type 2 diabetes. Our study also found that at different follow-up sessions, both sexes in the mHealth group reported significantly lower mean values of HbA_1c_, FBG, and P2BG. Patients aged 36-74 years in the mHealth group had steadily lower HbA_1c_, FBG, and P2BG mean values than those in the usual care group, and patients younger than 35 or older than 75 years old in the mHealth group reported unstable variation trends of mean values. There may be many reasons for poor glycemic control in patients older than 75 years of age in the mHealth group, including decreased self-management ability, inadequate exercise, irregular glycemic monitoring, and poor convenience in using apps, among other reasons [21,23,43]. For patients younger than 35 years old, the main reason may be poor compliance of patients and insufficient understanding of the importance of glycemic monitoring [24,25]. Especially for young patients, poor parental health literacy is the main reason [44,45].

However, some studies have found that even if blood glucose is effectively controlled, the occurrence and development of complications cannot be improved or reversed [46-48]. Researchers believe that this is due to the “metabolic memory” effect of hyperglycemia [46-48]. “Metabolic memory” effect refers to the persistent damage of early hyperglycemia to tissues and organs of diabetic patients, even though the glycemic control is good [48]. A growing body of experimental evidence supports the concept that the risk for diabetes complications may be linked to oxidative stress, nonenzymatic glycosylation of proteins, epigenetic changes, and chronic inflammation, laying the foundation for the “metabolic memory” theory [46]. From a clinical standpoint, the “metabolic memory” theory supports the need for very early aggressive treatment, with the goal of normalizing metabolic control as soon as possible, especially blood glucose. Therefore, achieving glycemic control targets as soon as possible and maintaining glycemic control for a long time have significantly positive effects in the prevention of complications [14]. The treatment strategy of diabetes should be changed from strict glycemic control to strict glycemic control at the early stage. The mobile-based intervention in this study seems to offer a promising option to implement this strategy. One meta-analysis of 35 randomized controlled trials found that an internet-based or mobile-based intervention duration of 3 months or less yielded optimal performance [49]. Our study also found that the glycemic control rates of the mHealth group were higher than those of the usual care group at the 3-, 6-, 9-, and 12-month follow-ups. These findings were not only consistent with previous studies, but also illustrated that the mobile-based intervention had generated a statistically significant improvement on glycemic control in the short and long term [47,49]. Therefore, implementation of mobile-based interventions could be a promising strategy for glycemic control of patients with diabetes not only at the early stage, but also in the long term.

In our study, the mobile-based intervention was designed to provide continuous, real-time, personalized health care for patients with diabetes, and it was delivered by a multidisciplinary team consisting of doctors, nurses, health educators, and dietitians. With the help of mobile technologies, this intervention provides a solution for diabetes management that includes the following: (1) a simple and intuitive way of vital data collection, (2) automatic in-hospital exam data and at-home data consolidation, (3) convenient and timely communication with care team professionals, and (4) continuous and vivid diabetes education, both in person and through multimedia. Based on the hardware equipment and professional support team, we have realized real-time guidance and management for diabetic patients; meanwhile, we have also collected a large amount of sample data. These data from the real world reflected the effectiveness of this mobile-based intervention. Notably, we found that, starting from the sixth month, the glycemic control of patients with type 2 diabetes in the mHealth group began to fluctuate slightly. This is a reminder that intensive management needs to be conducted to maintain the long-term effectiveness of this mobile-based intervention from the sixth month.

### Strengths and Limitations

A major strength of this study was the high-quality, continuously updated, clinical database of electronic medical records that provided a large sample size and reflected real-world clinical conditions. In addition, in our study, propensity score matching was used to control the confounding factors between the two groups. Propensity score matching could reduce the bias resulting from confounding variables; this approach attempted to mimic randomization by creating a sample of units that received the mobile-based intervention that is comparable, on all observed covariates, to a sample of units that received usual care.

This study had several limitations. First, as a result of its retrospective nature, we may not have addressed unobserved confounders in propensity score matching. Therefore, selection bias may exist in this research. For this reason, we used the propensity score matching to balance the common available covariates of the two groups, including sex, age, comorbidity (ie, hyperlipidemia and hypertension), HbA_1c_ level, and LDL cholesterol level. Second, there are inherent limitations as to what data are recorded in the clinical medical records. For instance, the clinical medical records of patients in the usual care group did not include some demographic information, such as education level, economic level, and occupation, among others, as well as anthropometry data, such as height, weight, systolic blood pressure, and diastolic blood pressure, among others. Cognitive function was not evaluated for either group. Third, in this study, the total proportion of missing values was 12.3%, and the missing values were on continuous variables, including HbA_1c_, FBG, P2BG, and LDL cholesterol. In order to decrease the amount of bias in the data, we used expectation maximization to estimate the missing values of continuous variables.

### Conclusions

This mobile-based intervention delivered by a multidisciplinary team to promote glycemic control of patients with type 2 diabetes led to increases in the control rates of HbA_1c_, FBG, and P2BG. These effects were best sustained within the first 6 months. It is noteworthy that, starting from the sixth month, intensive management might need to be conducted to maintain long-term effectiveness of this mobile-based intervention.

## Appendix

Multimedia Appendix 1 In-clinic user flow of the mHealth group.

Multimedia Appendix 2 Standardized differences before and after propensity score matching.

Multimedia Appendix 3 Variation trends of glycated hemoglobin (HbA1c), fasting blood glucose (FBG), and postprandial 2-hour blood glucose (P2BG) mean values (sex groups).

Multimedia Appendix 4 Variation trends of glycated hemoglobin (HbA1c), fasting blood glucose (FBG), and postprandial 2-hour blood glucose (P2BG) mean values (age groups).

Multimedia Appendix 5 Subgroup analysis of glycated hemoglobin (HbA1c) (%) between usual care and mHealth groups.

Multimedia Appendix 6 Subgroup analysis of fasting blood glucose (FBG) (mmol/L) between usual care and mHealth groups.

Multimedia Appendix 7 Subgroup analysis of postprandial 2-hour blood glucose (P2BG) (mmol/L) between usual care and mHealth groups.

## Abbreviations

FBG: fasting blood glucose
HbA_1c_: glycated hemoglobin
LDL: low-density lipoprotein
mHealth: mobile health
P2BG: postprandial 2-hour blood glucose
VTD: variation trends of difference
