# Supplementary material for: A Mobile-Based Intervention for Glycemic Control in Patients With Type 2 Diabetes: Retrospective, Propensity Score-Matched Cohort Study
Source: JMIR Mhealth Uhealth. 2020 Mar 11;8(3):e15390. doi: 10.2196/15390 (PMC7097724; doi:10.2196/15390)
Supplement: Multimedia Appendix 1 [file mhealth_v8i3e15390_app1.doc]

Multimedia Appendix 1. In-clinic user flow of mHealth group.


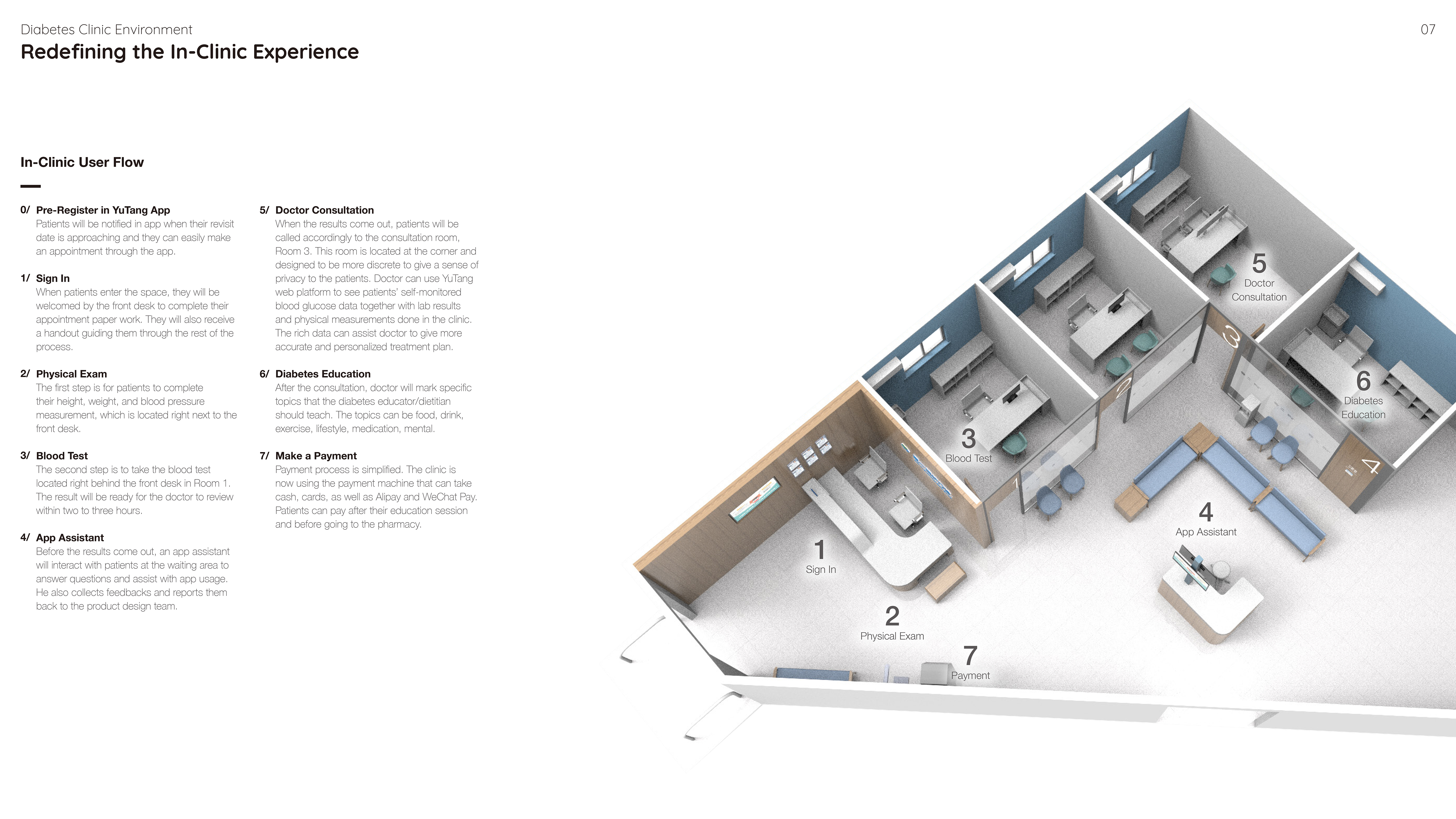


Patents will be notified in the app when their revisit date is approaching and they can easily make an appointment through the app. When patients enter the clinic, they will be welcomed by the front desk to complete their appointment paper work. They will also receive a handout guiding them through the rest of the process. The first step is for patient to complete their height, weight, and blood pressure measurement, which is located right next to the front desk. The second step is to take the blood test located right behind the front desk in one room. The result will be ready for the doctor to review within two to three hours. Before the results come out, an app assistant will interact with patients at the waiting area to answer questions and assist with app usage. When the results come out, patients will be called accordingly to be the consultation room. Doctor can use YuTang web platform to see patients’ self-monitored glycemic data together with lab results and physical measurements done in the clinic. These data can assist doctor to give more accurate and personalized treatment plan. After the consultation, doctor will mark specific topics that the diabetes educator/dietitian should teach. The topics can be food, drink, exercise, lifestyle, medication, and mental. The last step is for patient to pay after their education session and before going to the pharmacy.

The sharing of patient health data is the basis. With the help of YuTang cloud, patients’ data from all aspects (in hospital and outside of hospital) can now be consolidated. Medical professionals in the tertiary specialty hospital for diabetes, YuTang service support team, as well as doctors at the community hospitals can utilize this information to give individualized advice to patients.
