# Supplementary figures and images for: A Mobile-Based Intervention for Glycemic Control in Patients With Type 2 Diabetes: Retrospective, Propensity Score-Matched Cohort Study
Source: JMIR Mhealth Uhealth. 2020 Mar 11;8(3):e15390. doi: 10.2196/15390 (PMC7097724; doi:10.2196/15390)

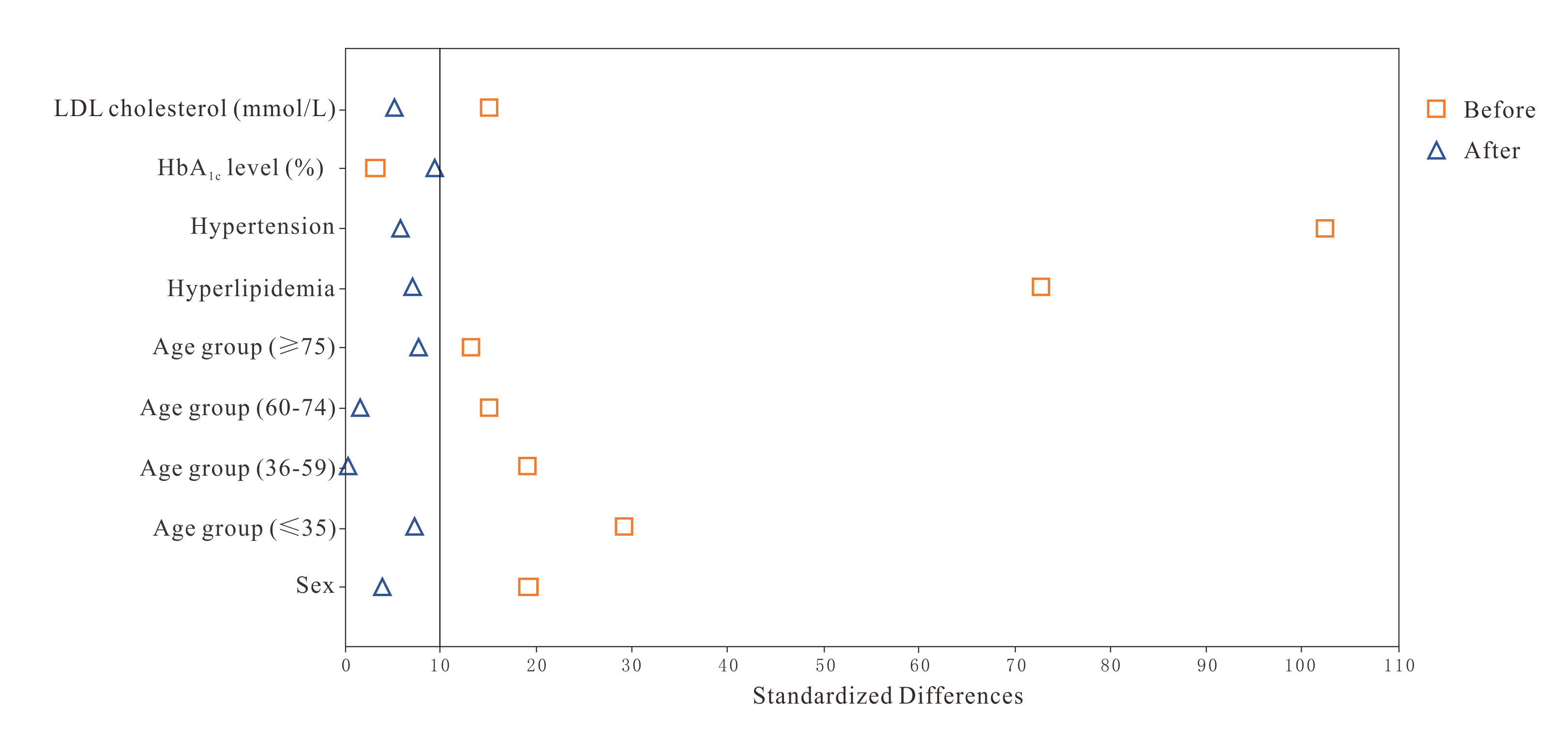


Multimedia Appendix 2. Standardized differences before and after match.

Supplement: Multimedia Appendix 2 [file mhealth_v8i3e15390_app2.doc]

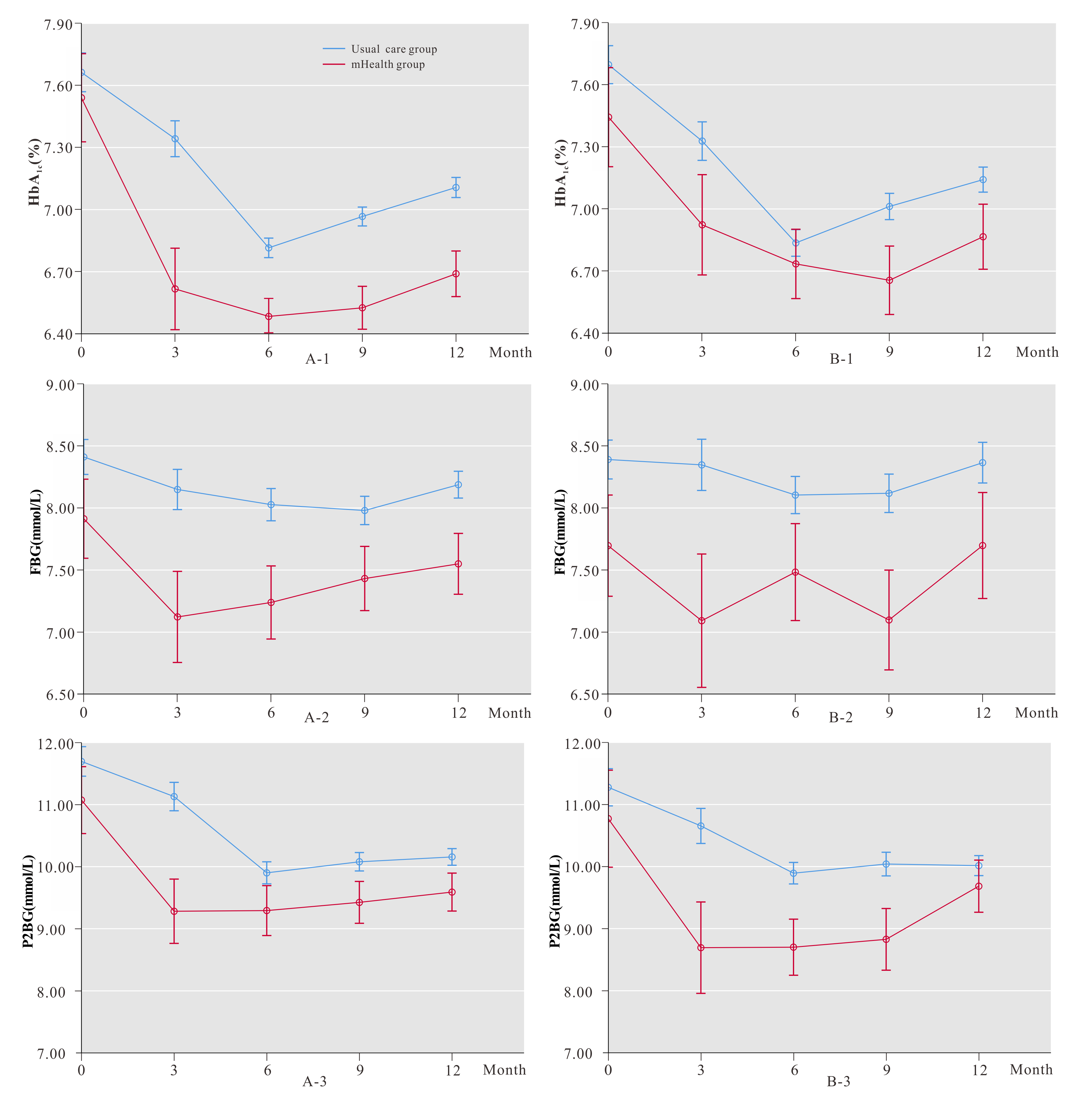


Multimedia Appendix 3. Variation trends of HbA1c, FBG, and P2BG means. (A) male; (B) female.

Supplement: Multimedia Appendix 3 [file mhealth_v8i3e15390_app3.doc]
