# Supplementary material for: A Mobile-Based Intervention for Glycemic Control in Patients With Type 2 Diabetes: Retrospective, Propensity Score-Matched Cohort Study
Source: JMIR Mhealth Uhealth. 2020 Mar 11;8(3):e15390. doi: 10.2196/15390 (PMC7097724; doi:10.2196/15390)
Supplement: Multimedia Appendix 4 [file mhealth_v8i3e15390_app4.doc]

**
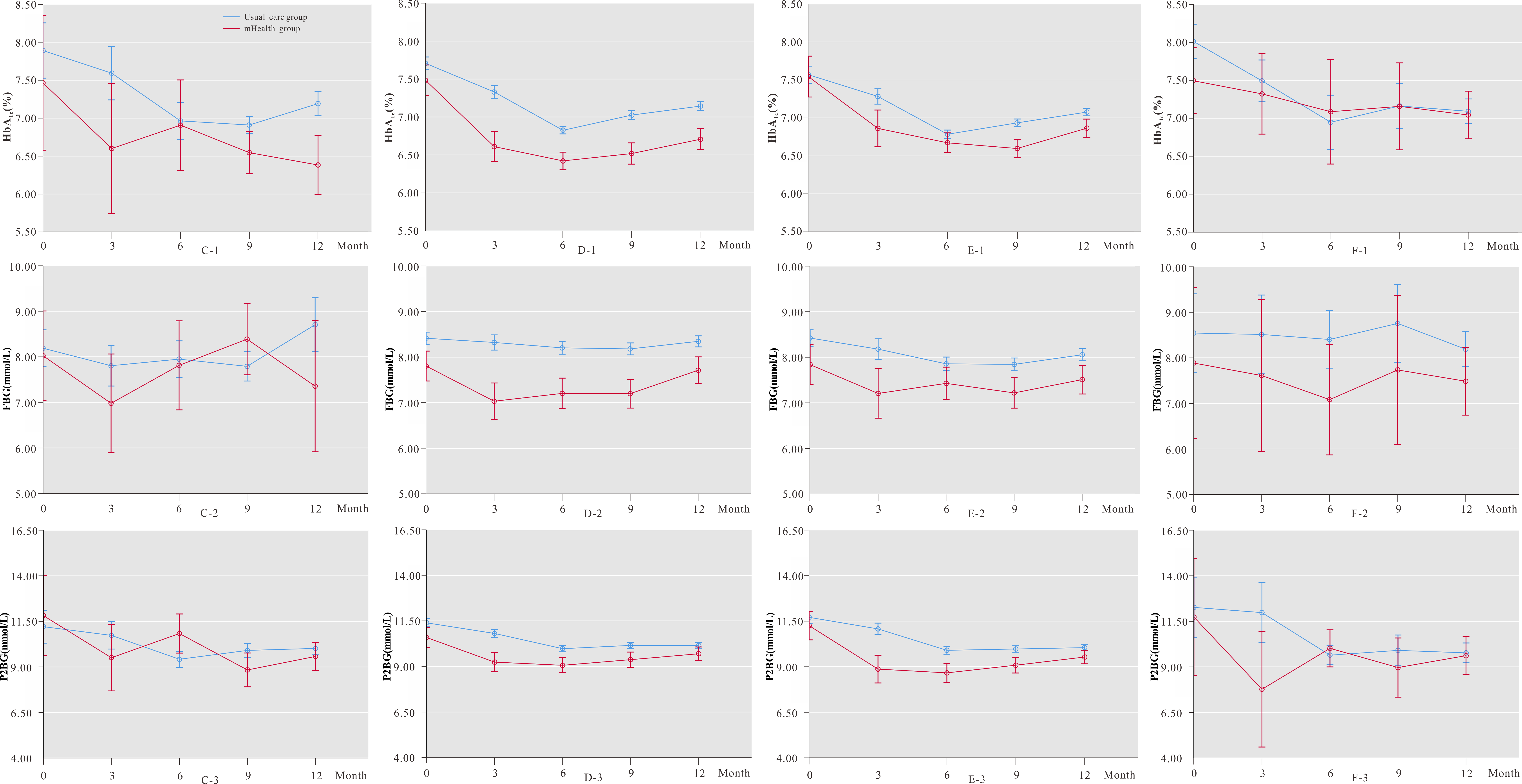
**

Multimedia Appendix 4. Variation trends of HbA_1c_, FBG, and P2BG means. (C) ≤ 35 years old; (D) 36-59 years old; (E) 60-74 years old; (F) ≥ 75 years old.
