# Supplementary material for: A Mobile-Based Intervention for Glycemic Control in Patients With Type 2 Diabetes: Retrospective, Propensity Score-Matched Cohort Study
Source: JMIR Mhealth Uhealth. 2020 Mar 11;8(3):e15390. doi: 10.2196/15390 (PMC7097724; doi:10.2196/15390)
Supplement: Multimedia Appendix 5 [file mhealth_v8i3e15390_app5.doc]

Multimedia Appendix 5. Subgroup analysis of HbA_1c_ (%) between usual care and mHealth groups.

|  | 3 months | | |  | 6 months | | |  | 9 months | | |  | 12 months | | |
| --- | --- | --- | --- | --- | --- | --- | --- | --- | --- | --- | --- | --- | --- | --- | --- |
| Characteristic | mHealth group | Usual care group | *P* value |  | mHealth group | Usual care group | *P* value |  | mHealth group | Usual care group | *P* value |  | mHealth group | Usual care group | *P* value |
|  |  |  |
| Sex, Mean (SD) |  |  |  |  |  |  |  |  |  |  |  |  |  |  |  |
| Male | 6.62 (0.78) | 7.34 (1.20) | <.001 |  | 6.48 (0.77) | 6.82 (0.58) | <.001 |  | 6.53 (0.78) | 6.97 (0.56) | <.001 |  | 6.70 (0.78) | 7.11 (0.61) | <.001 |
| Female | 6.92 (0.90) | 7.33 (1.07) | <.001 |  | 6.73 (0.70) | 6.84 (0.73) | <.001 |  | 6.66 (0.73) | 7.01 (0.71) | <.001 |  | 6.87 (0.70) | 7.14 (0.68) | <.001 |
| Age group (years), Mean (SD) | |  |  |  |  |  |  |  |  |  |  |  |  |  |  |
| ≤ 35 | 6.60 (1.01) | 7.59 (1.76) | <.001 |  | 6.91 (1.22) | 6.96 (1.15) | <.001 |  | 6.54 (0.81) | 6.91 (0.49) | <.001 |  | 6.38 (0.64) | 7.19 (0.78) | <.001 |
| 36-59 | 6.61 (0.87) | 7.33 (1.06) | <.001 |  | 6.42 (0.71) | 6.83 (0.59) | <.001 |  | 6.52 (0.83) | 7.03 (0.71) | <.001 |  | 6.71 (0.82) | 7.14 (0.71) | <.001 |
| 60-74 | 6.86 (0.72) | 7.28 (1.11) | <.001 |  | 6.67 (0.61) | 6.79 (0.57) | <.001 |  | 6.60 (0.66) | 6.93 (0.51) | <.001 |  | 6.87 (0.65) | 7.08 (0.50) | .001 |
| ≥ 75 | 7.32 (0.65) | 7.49 (0.70) | .002 |  | 7.09 (1.12) | 6.95 (0.83) | .023 |  | 7.16 (0.41) | 7.16 (0.80) | .126 |  | 7.04 (0.89) | 7.09 (0.12) | .916 |
| Comorbidity, Mean (SD) | |  |  |  |  |  |  |  |  |  |  |  |  |  |  |
| Hyperlipidemia | 6.76 (0.70) | 7.45 (1.25) | <.001 |  | 6.61 (0.72) | 6.81 (0.58) | <.001 |  | 6.61 (0.68) | 6.98 (0.62) | <.001 |  | 6.75 (0.68) | 7.12 (0.63) | <.001 |
| Hypertension | 6.70 (0.80) | 7.24 (1.08) | <.001 |  | 6.60 (0.81) | 6.79 (0.52) | <.001 |  | 6.60 (0.74) | 6.99 (0.63) | <.001 |  | 6.77 (0.72) | 7.06 (0.48) | <.001 |
