# Supplementary material for: A Mobile-Based Intervention for Glycemic Control in Patients With Type 2 Diabetes: Retrospective, Propensity Score-Matched Cohort Study
Source: JMIR Mhealth Uhealth. 2020 Mar 11;8(3):e15390. doi: 10.2196/15390 (PMC7097724; doi:10.2196/15390)
Supplement: Multimedia Appendix 6 [file mhealth_v8i3e15390_app6.doc]

Multimedia Appendix 6. Subgroup analysis of FBG (mmol/L) between usual care and mHealth groups.

|  | 3 months | | |  | 6 months | | |  | 9 months | | |  | 12 months | | |
| --- | --- | --- | --- | --- | --- | --- | --- | --- | --- | --- | --- | --- | --- | --- | --- |
| Characteristic | mHealth group | Usual care group | *P* value |  | mHealth group | Usual care group | *P* value |  | mHealth group | Usual care group | *P* value |  | mHealth group | Usual care group | *P* value |
|  |  |  |
| Sex, Mean (SD) |  |  |  |  |  |  |  |  |  |  |  |  |  |  |  |
| Male | 7.12 (1.22) | 8.15 (2.26) | <.001 |  | 7.24 (1.49) | 8.03 (1.74) | <.001 |  | 7.43 (1.59) | 7.98 (1.48) | <.001 |  | 7.55 (1.21) | 8.19 (1.45) | <.001 |
| Female | 7.09 (1.39) | 8.35 (2.43) | <.001 |  | 7.48 (1.58) | 8.10 (1.70) | <.001 |  | 7.10 (1.16) | 8.12 (1.81) | <.001 |  | 7.70 (0.98) | 8.37 (1.94) | .027 |
| Age group (years), Mean (SD) | |  |  |  |  |  |  |  |  |  |  |  |  |  |  |
| ≤ 35 | 6.98 (1.39) | 7.80 (2.21) | .063 |  | 7.81 (2.89) | 7.95 (1.70) | .002 |  | 8.39 (2.44) | 7.79 (1.33) | .344 |  | 7.36 (1.00) | 8.71 (3.00) | .001 |
| 36-59 | 7.03 (1.29) | 8.32 (2.20) | <.001 |  | 7.20 (1.21) | 8.20 (1.82) | <.001 |  | 7.19 (1.13) | 8.18 (1.73) | <.001 |  | 7.71 (1.17) | 8.35 (1.58) | <.001 |
| 60-74 | 7.21 (1.25) | 8.18 (2.52) | <.001 |  | 7.43 (1.51) | 7.86 (1.57) | <.001 |  | 7.22 (1.53) | 7.84 (1.46) | <.001 |  | 7.51 (1.10) | 8.06 (1.43) | .005 |
| ≥ 75 | 7.61 (1.30) | 8.51 (2.32) | .043 |  | 7.08 (1.95) | 8.40 (1.47) | <.001 |  | 7.73 (1.83) | 8.75 (2.19) | .033 |  | 7.49 (1.04) | 8.19 (0.95) | .156 |
| Comorbidity, Mean (SD) | |  |  |  |  |  |  |  |  |  |  |  |  |  |  |
| Hyperlipidemia | 7.26 (1.23) | 8.24 (2.38) | <.001 |  | 7.63 (1.46) | 7.89 (1.54) | <.001 |  | 7.24 (1.08) | 7.92 (1.52) | <.001 |  | 7.63 (0.87) | 8.11 (1.45) | .002 |
| Hypertension | 7.05 (1.24) | 8.20 (2.27) | <.001 |  | 7.19 (1.47) | 7.91 (1.45) | <.001 |  | 7.25 (1.63) | 7.98 (1.59) | <.001 |  | 7.49 (1.18) | 8.16 (1.67) | .002 |
