# Supplementary material for: A Mobile-Based Intervention for Glycemic Control in Patients With Type 2 Diabetes: Retrospective, Propensity Score-Matched Cohort Study
Source: JMIR Mhealth Uhealth. 2020 Mar 11;8(3):e15390. doi: 10.2196/15390 (PMC7097724; doi:10.2196/15390)
Supplement: Multimedia Appendix 7 [file mhealth_v8i3e15390_app7.doc]

Multimedia Appendix 7. Subgroup analysis of P2BG (mmol/L) between usual care and mHealth groups.

|  | 3 months | | |  | 6 months | | |  | 9 months | | |  | 12 months | | |
| --- | --- | --- | --- | --- | --- | --- | --- | --- | --- | --- | --- | --- | --- | --- | --- |
| Characteristic | mHealth group | Usual care group | *P* value |  | mHealth group | Usual care group | *P* value |  | mHealth group | Usual care group | *P* value |  | mHealth group | Usual care group | *P* value |
|  |  |  |
| Sex, Mean (SD) |  |  |  |  |  |  |  |  |  |  |  |  |  |  |  |
| Male | 9.28 (2.12) | 11.13 (3.14) | <.001 |  | 9.29 (2.11) | 9.90 (2.37) | <.001 |  | 9.42 (1.88) | 10.08 (1.97) | <.001 |  | 9.59 (1.56) | 10.16(1.80) | .002 |
| Female | 8.69 (2.40) | 10.65 (3.28) | <.001 |  | 8.70 (1.75) | 9.89 (1.98) | <.001 |  | 8.83 (1.80) | 10.04 (2.19) | <.001 |  | 9.68 (1.37) | 10.01(1.87) | .200 |
| Age group (years), Mean (SD) | |  |  |  |  |  |  |  |  |  |  |  |  |  |  |
| ≤ 35 | 9.51 (3.22) | 10.73(3.61) | <.001 |  | 10.83 (4.41) | 9.42 (1.41) | <.001 |  | 8.84 (1.60) | 9.91 (1.84) | <.001 |  | 9.58 (1.29) | 10.02(1.53) | .235 |
| 36-59 | 9.24 (1.94) | 10.81(2.87) | <.001 |  | 9.07 (1.59) | 9.98 (2.22) | <.001 |  | 9.37 (1.69) | 10.16 (2.24) | <.001 |  | 9.70 (1.53) | 10.16(2.00) | .092 |
| 60-74 | 8.87 (2.35) | 11.07(3.48) | <.001 |  | 8.66 (1.58) | 9.90 (2.37) | <.001 |  | 9.08 (2.08) | 9.97 (1.83) | <.001 |  | 9.53 (1.53) | 10.05(1.65) | .014 |
| ≥ 75 | 7.77 (2.53) | 11.98(4.40) | <.001 |  | 10.02(1.77) | 9.64 (1.19) | .002 |  | 8.96 (2.67) | 9.90 (1.95) | .053 |  | 9.61 (0.94) | 9.77 (1.43) | .449 |
| Comorbidity, Mean (SD) | |  |  |  |  |  |  |  |  |  |  |  |  |  |  |
| Hyperlipidemia | 9.17 (2.03) | 11.12(3.36) | <.001 |  | 8.89 (1.62) | 9.94 (1.93) | <.001 |  | 9.20 (1.93) | 10.01(1.78) | <.001 |  | 9.73 (1.30) | 10.14(1.93) | .055 |
| Hypertension | 9.03 (2.37) | 10.94(3.08) | <.001 |  | 8.89 (1.42) | 9.91 (1.83) | <.001 |  | 9.35 (2.04) | 10.05 (1.69) | <.001 |  | 9.56 (1.34) | 10.07(1.63) | .010 |
